# Anticancer Effect of *Cycas media*: Molecular Basis Through Modulation of PI3K/AKT/mTOR Signaling Pathway

**DOI:** 10.3390/molecules29215013

**Published:** 2024-10-23

**Authors:** Jawaher Alqahtani, Esraa M. Mosalam, Hend E. Abo Mansour, Aya Ibrahim Elberri, Hanaa A. Ibrahim, Sebaey Mahgoub, Ismail A. Hussein, Mohammed F. Hawwal, Maryam Al Hmoudi, Ehssan Moglad, Rehab Ahmed, Fatma Alzahraa Mokhtar, Engy Elekhnawy, Walaa A. Negm

**Affiliations:** 1 Department of Pharmacognosy, College of Pharmacy, King Saud University, Riyadh 11495, Saudi Arabia; mhawwal@ksu.edu.sa; 2 Biochemistry Department, Faculty of Pharmacy, Menoufia University, Shebin El-Kom 32511, Egypt; hend_elsaid@phrm.menofia.edu.eg; 3 Department of Pharm D, Faculty of Pharmacy, Jadara University, Irbid 21110, Jordan; 4 Biochemistry Department, Faculty of Pharmacy, Menoufia National University, Birket El-Sab 32651, Egypt; 5 Genetic Engineering and Molecular Biology Division, Department of Zoology, Faculty of Science, Menoufia University, Shebin El-Kom 32511, Egypt; ayaelbery62@science.menofia.edu.eg; 6 Department of Pharmacology and Toxicology, Faculty of Pharmacy, Tanta University, Tanta 31527, Egypt; hanaa.abdelkareem@pharm.tanta.edu.eg; 7 Food Analysis Laboratory, Ministry of Health, Zagazig 44511, Egypt; dr_semahgoub@yahoo.com; 8 Department of Pharmacognosy and Medicinal Plants, Faculty of Pharmacy (Boys), Al-Azhar University, Cairo 11884, Egypt; ismaila.hussein@azhar.edu.eg; 9 Fujairah Research Centre, Sakamkam Road, Fujairah 00000, United Arab Emirates; 10 Department of Pharmaceutics, College of Pharmacy, Prince Sattam bin Abdulaziz University, Alkharj 11942, Saudi Arabia; e.moglad@psau.edu.sa; 11 Department of Natural Products and Alternative Medicine, Faculty of Pharmacy, University of Tabuk, Tabuk 47713, Saudi Arabia; rahmed@ut.edu.sa; 12 Department of Pharmacognosy, Faculty of Pharmacy, El Saleheya El Gadida University, El Saleheya El Gadida 44813, Egypt; drfatmaalzahraa1950@gmail.com; 13 Pharmaceutical Microbiology Department, Faculty of Pharmacy, Tanta University, Tanta 31527, Egypt; 14 Department of Pharmacognosy, Faculty of Pharmacy, Tanta University, Tanta 31527, Egypt

**Keywords:** *Cycas media*, HepG2, EAC, antitumor, PI3K/AKT/mTOR, *Staphylococcus aureus*

## Abstract

Many researchers are focusing on screening the biological activities of plants owing to their safety and possible pharmacological actions. Consequently, we aimed to explore the antiproliferative and cytotoxic properties of *Cycas media* methanolic extract on HepG2 cell lines. Moreover, we also explore the antitumor action against the experimentally induced solid Ehrlich carcinoma (SEC) model and investigate the possible involved molecular mechanisms. Also, the antibacterial action of the extract was elucidated. Different concentrations of the extract were incubated with HepG2 to determine cytotoxicity, followed by cell cycle analysis. The in vivo experiment was accomplished by grouping the animals into four different groups (*n* = 10); normal control, SEC, *C. media* 100, and *C. media* 200. The extract was administered at 100 and 200 mg/kg. Tumor volume, tumor inhibition rate, toxicity profile, and antioxidant biomarkers were determined. Moreover, the PI3K/AKT/mTOR signaling pathway was investigated as a possible underlying antitumor mechanism. The tumor control group showed a remarkable upregulation for PI3K, p-AKT, and p-mTOR, along with downregulation for the antioxidant SOD and GPX4, as well as decreased levels of GSH and MDA. *C. media* extract reversed these parameters to a significant level and the higher dose showed a superior antitumor effect. *C. media* extract showed antiproliferative effects against HepG2 cells, along with a suppressive action on the PI3K/AKT/mTOR pathway and an antioxidant effect. Additionally, *C. media* had antibacterial consequences against *S. aureus* isolates with minimum inhibitory concentrations from 32 to 128 µg/mL. It also caused a noteworthy growth delay as well as a notable reduction in the membrane integrity of *S. aureus* isolates. These beneficial outcomes suggest *C. media* to have potential antitumor and antibacterial activities.

## 1. Introduction

An adverse environment is created for healthy cells by the aberrant modern lifestyle such as smoking and improper eating habits. This leads to the dysregulation of cell growth and results in uncontrolled cell proliferation of abnormal cells known as cancer cells, which can generate tumors and invade other tissues [1]. There are over 277 distinct cancers with varying genetics worldwide, and these numbers are expected to rise significantly in the next few decades [2]. The complicated pathophysiology and genetic variations of cancer provide challenges for pharmaceutical regimens. Moreover, the severe side effects of cancer chemotherapeutics significantly impact the quality of patients’ lives. These issues could be addressed by developing novel therapeutics [3].

Global recognition for plant-based medicine is growing due to its superior safety record. The direction of the current research is toward drug development based on natural products and linking it to pharmacology and combinatorial chemistry [4,5]. *Cycas media* from *Cycadaceae* have short stems and grow like palm trees. For millennia, people have been aware of *Cycas* plants, which are found in tropical areas [6]. Pharmacological research has demonstrated the plant’s antibacterial, antifungal, antiulcer, and antitumor properties [7,8,9]. A variety of polyphenolic chemicals including flavonoids, terpenoids, lignans, monolignols, aromatic acids, and sterols, are abundant in the species of the *Cycas* genus [10,11]. The flavan nucleus distinguishes flavonoids as the main active ingredients in the genus *Cycas*. Flavanones, flavones, isoflavones, and biflavonoids are the most common structural kinds of flavonoids. Furthermore, terpenoids are a broad class of naturally occurring compounds that are generated from C5 isoprene units. Additionally, lignans are secondary metabolites that are also polyphenols and they are produced when phenyl propenes polymerize [12,13]. Preceding studies also showed that other *Cycas* species have antitumor activity against different tumorigenic cells such as hepatic, breast, and colon cancerous cells [7,14,15].

Phosphatidylinositol 3-kinase (PI3K)/protein kinase B (AKT)/mammalian target of rapamycin (mTOR) pathway possesses a fundamental role in various cellular functions. When a specific ligand binds to the corresponding cell-surface receptor, PI3k is activated, which in turn, activates the downstream effectors, involving AKT and mTOR [16]. PI3K/AKT/mTOR signaling pathway is involved in the activation of glucose metabolism, protein synthesis, and anti-apoptotic mechanisms. In order to preserve cellular homeostasis, mTOR regulates cellular metabolism, catabolism, immunological responses, autophagy, survival, proliferation, and migration. Three different multi-subunit complexes called mTOR complex 1/2/3 (mTORC1/2/3) make up the mTOR signaling cascade [17,18]. It was demonstrated that triggering the mTOR cascade increases tumor development via controlling angiogenesis, lipid metabolism, glycolysis, growth factor receptor pathway, and autophagy. Consequently, mTOR is a significant and intriguing target for therapeutic intervention against cancer [19,20,21]. These processes affect all cell growth and proliferation during tumor development [22]. Moreover, it was found that there is a crosstalk between PI3K/AKT/mTOR signaling and the oxidative stress [23]. Reactive oxygen species (ROS) that are released by oxidative stress can induce a pathogenic state because they cause DNA damage by triggering an inflammatory response driven by oxidative stress. Fortunately, the antioxidant capacity of the cells can be boosted through upregulation of the endogenous antioxidants such as glutathione peroxidase (GPX) and superoxide dismutase (SOD) [6].

Bacterial infections are frequently prevalent among immunocompromised patients like those who suffer from cancer. They usually use antibiotics to treat such infections, but the failure of the antibiotics commonly arises among these patients and this escalates the occurrence of sepsis and subsequently death [24]. *Staphylococcus aureus*, a Gram-positive bacterium, frequently predisposes infections in cancer patients as it has the ability to colonize and invade human tissues. It possesses many virulent factors in addition to its great capacity to resist many antibiotics. Thus, it is important to develop novel antibacterials against *S. aureus* isolates to decrease the incidence of death among infected cancer patients [25].

In this context, this research was designed to explore the antiproliferative and cytotoxic properties of *C. media* methanolic extract on human HepG2 cell lines. Moreover, we explore the in vivo antitumor action against experimentally induced mammary carcinoma in an animal model and investigate the possible involved molecular mechanisms. Also, the antibacterial activity of *C. media* was assessed against the *S. aureus* clinical bacteria isolated from cancer patients.

## 2. Results

### 2.1. Phytochemical Elucidation of C. media by LC–ESI–MS/MS

**Figure 1:**
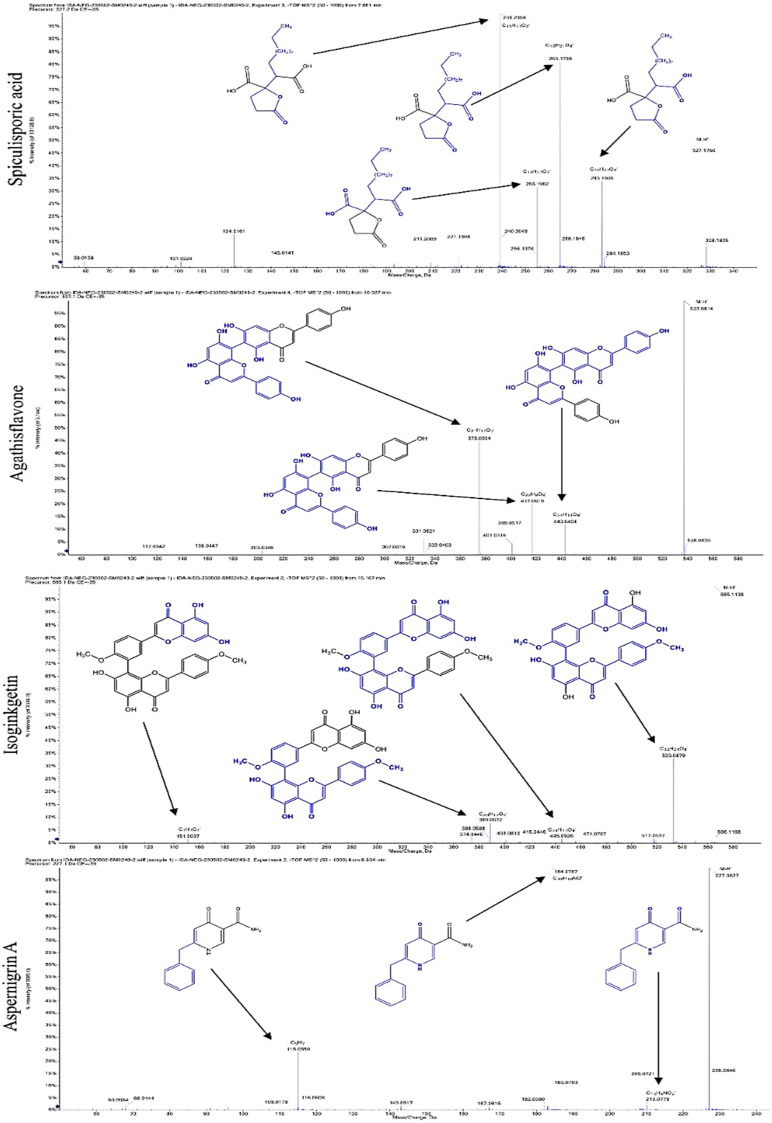
Mass/mass spectra showed the fragmentation pattern of major identified compounds.

The LC–MS/MS technique tentatively revealed that *C. media* possessed many phytochemical secondary metabolites. Table 1 demonstrates the tentatively documented 33 compounds reinforced with referenced data. Figure 1 displays mass/mass spectra that reveal the pattern of key detected metabolites’ fragmentation.

### 2.2. Effect on Cytotoxicity

**Figure 2:**
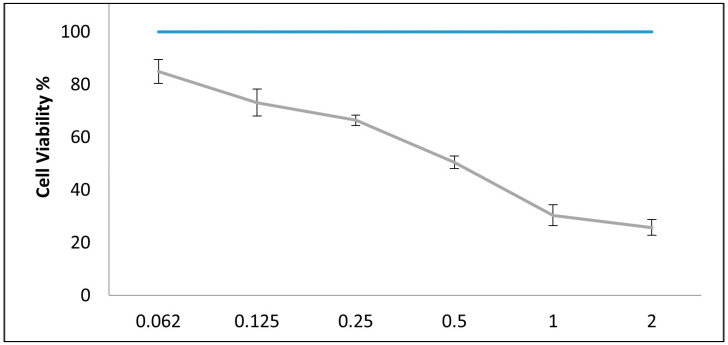
Cellular viability of HepG2 cells exposed to 2, 1, 0.5, 0.25, 0.125, and 0.0625 mg/mL of *C. media* extract. The IC_50_ value was found to be 0.5183 mg/mL calculated from 3 repeats.

The *C. media*’s anticancer impact on HepG2 cells was assessed using the micro-culture-tetrazolium test. The viability of HepG2 cells was diminished with increasing concentration when they were exposed to a serial concentration of *C. media* extract for 24 h (2, 1, 0.5, 0.25, 0.125, and 0.0625 mg/mL). The viability of the cells exposed to the *C. media* extract at concentrations 2, 1, 0.5, 0.25, 0.125, and 0.0625 mg/mL was found to be 84.9%, 73.1%, 66.4%, 50.4%, 30.3%, and 25.7%, respectively. The HepG2 cells were significantly inhibited by the methanol extract of *C. media*, with an IC_50_ value equal to 0.5183 mg/mL (Figure 2).

### 2.3. Cell Cycle Analysis

**Figure 3:**
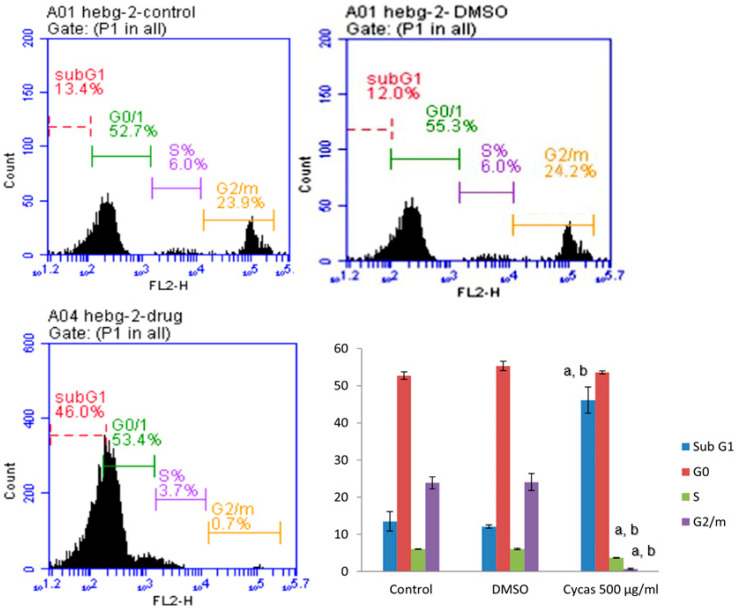
Effect of 500 µg/mL of *C. media* extract on cell cycle distribution of treated and control HepG2 cells. The cell cycle phases were analyzed after propidium iodide labeling. Data are presented as mean ± SD of three different experiments, (*p* < 0.01), a: significant versus control and b: significant versus DMSO.

*C. media* extract-treated cells with a concentration of 500 µg/mL exhibited unusual DNA fashion with sub-G1, G0, S and G2/M phases of the cell cycle. At the start, the treated cells revealed a greater sub-G1 population (46%) relative to the control (13.4%). *C. media* extract showed an accompanying decrease in the cellular population in the S and G2/M phases from 6% to 23.9%, respectively, compared to the control, with 3.7% and 0.7%, respectively (Figure 3). The proportion of the sub-G1 phase was substantially raised after the cells were treated with the extract in comparison with the control.

### 2.4. Tumor Volume and Tumor Inhibition Rate in Experimental Animals

**Figure 4:**
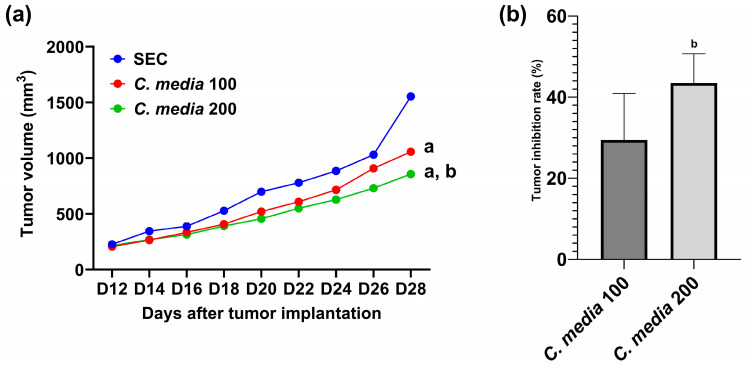
Effect of *C. media* extract on (**a**) tumor volume and (**b**) tumor inhibition rate in SEC-bearing mice. Data are presented as mean ± SD for 10 mice and were analyzed using one-way ANOVA followed by Tukey post hoc test. *p* < 0.05, a: significant versus tumor control and b: significant versus *C. media* extract 100. SEC: solid Ehrlich carcinoma.

After 12 days of Ehrlich ascites carcinoma (EAC) implementation, there was a remarkable escalation in the volume of tumors in all the studied groups, as presented in Figure 4. On day 28, a substantial lessening in the tumor volume was observed in the two groups treated with *C. media* extract but the higher dose (200 mg/kg) was more effective compared to the lower dose (100 mg/kg). Regarding tumor inhibition rate (TIR), there was a notable inhibition (29.46%) by *C. media* 100 and the higher dose inhibited the tumor by 43.5%, which was a significant difference (*p* = 0.011) compared to the lower dose (Figure 4).

### 2.5. In Vivo Toxicity Profile of C. media Extract

**Figure 5:**
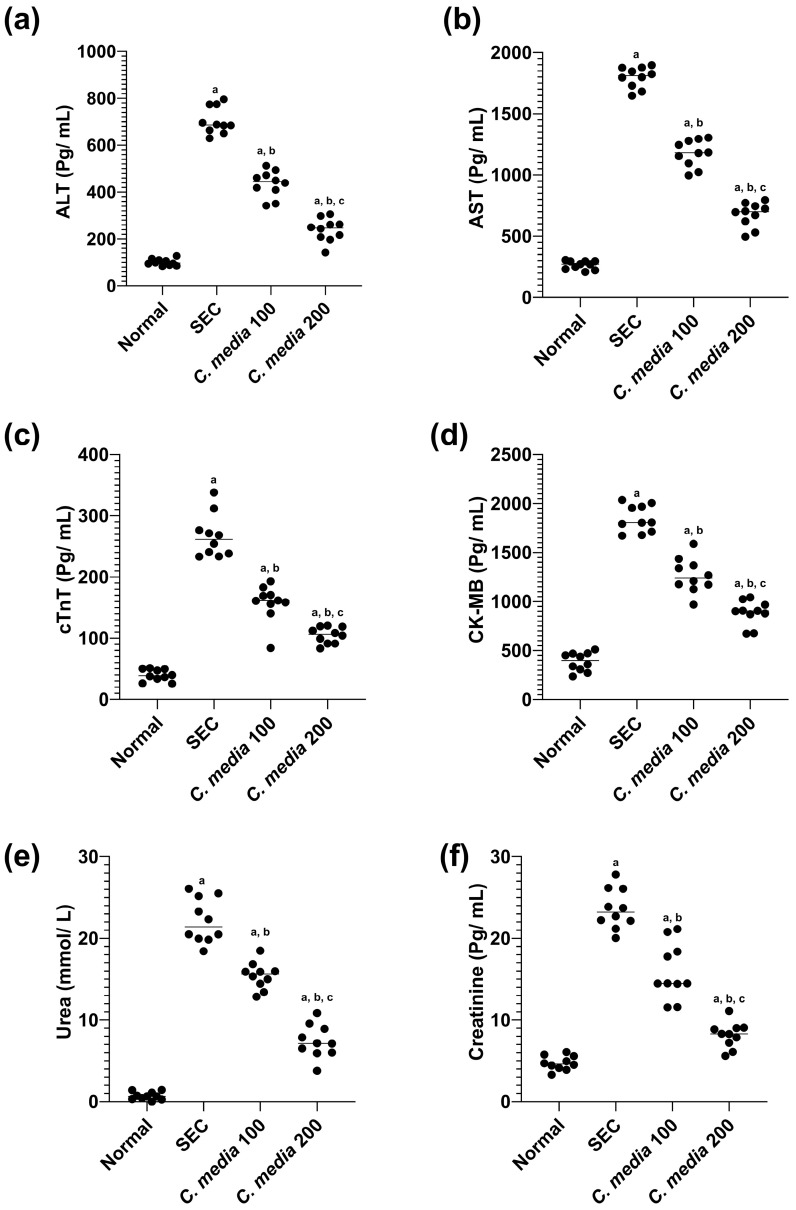
Toxicity profile of *C. media* extract on hepatic, cardiac, and renal biomarkers in SEC-bearing mice. (**a**) ALT, (**b**) AST, (**c**) cTnT, (**d**) CK-MB, (**e**) urea, and (**f**) creatinine. Data are presented as mean ± SD for 10 mice and were analyzed using one-way ANOVA followed by Tukey post hoc test. *p* < 0.05. a: significant versus normal control, b: significant versus disease control, c: significant versus *C. media* extract 100. SEC: solid Ehrlich carcinoma, ALT: alanine aminotransferase, AST: aspartate aminotransferase, cTnT: cardiac troponin, CK-MB: creatin kinase MB.

The solid Ehrlich carcinoma (SEC)-diseased group displayed a substantial (*p* < 0.001) increase in the serum level of ALT, AST, cTnT, CK-MB, urea, and creatinine compared to the normal group. On the contrary, the treatment of the mice with *C. media* extract at both concentrations significantly decreased the serum level of these biomarkers by 38.24, 34.56, 40.84, 31.35, 30.44, and 32.59%, respectively, for those treated with *C. media* 100, whereas by 66.12, 62.39, 60.68, 52, 66.74, and 65.49%, respectively, for those treated with *C. media* 200, as displayed in Figure 5.

### 2.6. Effect on Oxidative Stress Biomarkers

**Figure 6:**
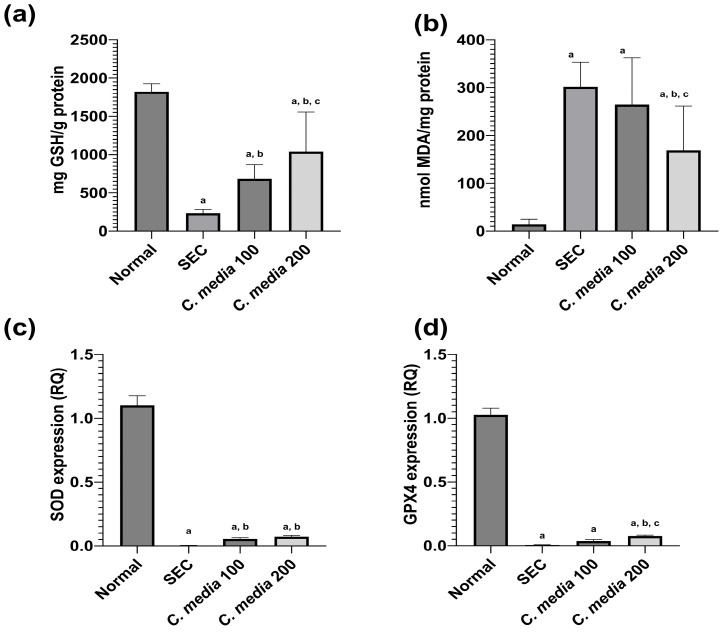
Effect of *C. media* extract on oxidative stress biomarkers and antioxidant enzymes in SEC-bearing mice. (**a**) GSH, (**b**) MDA, (**c**) SOD, and (**d**) GPX4. Data are presented as mean ± SD for 10 mice and were analyzed using one-way ANOVA followed by Tukey post hoc test. *p* < 0.05, a: significant versus normal control, b: significant versus disease control, and c: significant versus *C. media* extract 100. SEC: solid Ehrlich carcinoma, GSH: glutathione, MDA: malondialdehyde, SOD: superoxide dismutase, GPX4: glutathione peroxidase 4.

The SEC-diseased mice showed a noteworthy (*p* < 0.001) decline in the concentration of GSH along with a substantial (*p* < 0.001) rise in MDA concentration in comparison to the normal mice. Contrarily, the mice that were treated with *C. media* 100 exhibited a substantial rise (*p* = 0.005) in the level of GSH of 1.93 fold and a non-significant (*p* = 0.665) decline in the level of MDA by 12.27% compared to the untreated mice. Likewise, *C. media* 200-treated mice revealed a significant (*p* < 0.001) increase in the GSH content by 3.44 folds with a significant (*p* = 0.001) decline in the tumor content of MDA by 44.03% relative to the diseased mice, as shown in Figure 6.

Regarding the antioxidant enzymes, there was a noteworthy downregulation in the expression level of SOD and GPX4 (*p* < 0.001) in the SEC-diseased group compared to the normal. Administration of *C. media* 100 upregulated the genes for these two enzymes, which was significant only in the case of SOD against the diseased group. A similar effect was also observed in the mice that were treated with *C. media* 200, but it was significant (*p* < 0.01) for both genes compared to the untreated group (Figure 6).

### 2.7. Effect on PI3K/p-AkT/p-mTOR Signaling Pathway

**Figure 7:**
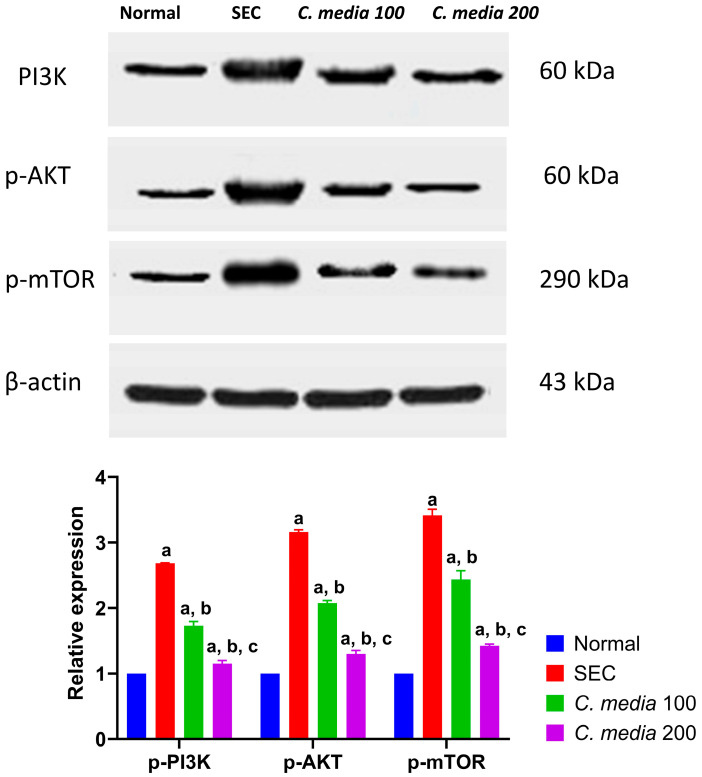
Modulation of PI3K/AKT/mTOR protein expression in the tumor tissue by *C. media* extract. Data are presented as mean ± SD for 3 readings per sample for 3 mice and were analyzed using one-way ANOVA followed by Tukey post hoc test. *p* < 0.05. a: significant versus normal control, b: significant versus disease control, c: significant versus *C. media* extract 100. SEC: solid Ehrlich carcinoma, PI3K: phosphatidylinositol 3-kinase, p-AKT: phosphorylated protein kinase B, p-mTOR: phosphorylated mammalian target of rapamycin.

Figure 7 shows that the SEC-diseased mice exhibited a noteworthy (*p* < 0.001) increase in the expression level of PI3K, p-AkT, and p-mTOR in the tumor tissue relative to the normal mice. Treatment of the mice with *C. media* 100 considerably (*p* < 0.001) downregulated these proteins by 35.57, 34.37, and 28.67%, respectively, compared to the diseased group. Similarly, mice treated with *C. media* 200 disclosed a substantial (*p* < 0.001) decline in the expression level of these proteins by 56.99, 58.94, and 58.28%, respectively, compared to the diseased group.

### 2.8. Bacterial Susceptibility to C. media

*C. media* revealed antibacterial potential using the cup plate method by triggering inhibition zones around the wells. Therefore, the MIC values were recorded using broth microdilution assay. *C. media* revealed MIC values from 32 to 128 µg/mL (Table 2).

### 2.9. Growth Kinetics

**Figure 8:**
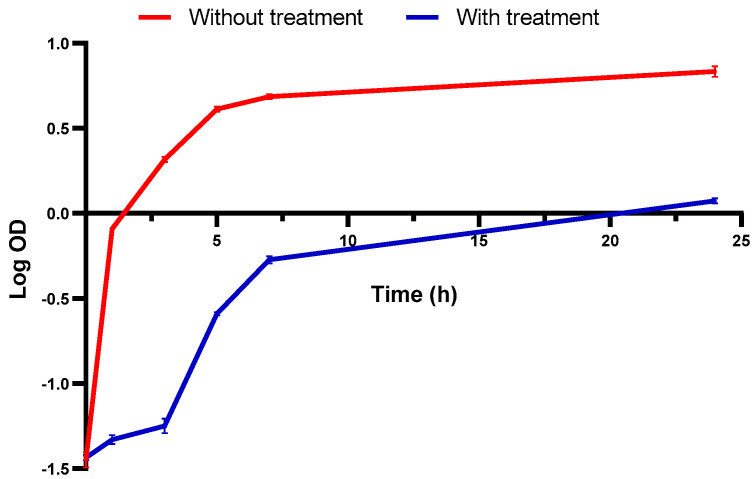
An illustrative graph for the influence of *C. media* on the growth kinetics of *S. aureus* isolates.

Growth curves were constructed for *S. aureus* isolates with and without *C. media* for studying its influence on growth kinetics. *C. media* revealed a noteworthy (*p* < 0.05) growth delay as illuminated in the illustrative curve (Figure 8).

### 2.10. Membrane Integrity

**Figure 9:**
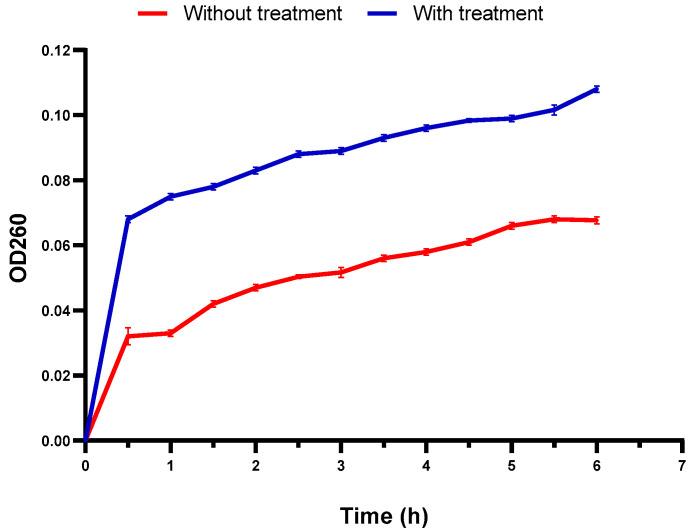
A descriptive diagram displaying the influence of *C. media* on the membrane integrity of *S. aureus* isolate.

It was examined with and without 0.5 MIC of *C. media*. Here, *C. media* exhibited a notable reduction (*p* < 0.05) of the integrity of the membrane in all the tested isolates. Figure 9 establishes a demonstrative illustration as the discharge of DNA and RNA was considerably greater (*p* < 0.05) in the presence of *C. media*. This indicates a significant decline (*p* < 0.05) in the integrity by *C. media*.

## 3. Discussion

Most plants possess significant pharmacological activities, which could aid in the prevention and treatment of numerous diseases. Therefore, screening for biological activities for different species has captured greater attention in recent decades [26,27]. To the best of our knowledge, this is the first study aimed at investigating the anticancer potential of *C. media* extract against HepG2 liver carcinoma cells and also against the EAC-induced mammary carcinoma model in mice.

Treatment of HepG2 cells with *C. media* extract showed an effective IC_50_ value of 0.5183 mg/mL in the cytotoxicity assay, along with a decreased cellular population in S and G2/M phases and an increased population in the apoptotic phase of the cell cycle, which indicates its anticancer activity. Our results are in harmony with previously published data that indicated the cytotoxic influence of different *Cycas* species [10,13].

Our in vivo molecular investigations were based on studying the antitumor consequences of *C. media* extract through modulation of the PI3K/AKT/mTOR signaling pathway, along with the possible effect on antioxidant defense mechanisms. The PI3K/AKT/mTOR axis plays a crucial role in metabolism, cell differentiation, cellular proliferation, cell cycle, apoptosis, and autophagy. When a specific ligand binds to the corresponding cell-surface receptor, PI3k is activated, which in turn, activates the downstream effectors including AKT and mTOR [16]. In some details, PI3K is a cell-membrane-bound enzyme that is responsible for the biosynthesis of some phospholipids such as phosphatidylinositol 4,5-bisphosphate (PIP2) and phosphatidylinositol 3,4,5-trisphosphate (PIP3). Consequently, AKT is phosphorylated by the recruitment of PIP2 and PIP3 at Thr308 and Ser473. mTOR is a serine/threonine kinase that integrates signals initiated by different growth factors and transmits them to regulate various cellular activities [22].

It was found that the PI3K/AKT/mTOR signaling pathway is associated with multiple types of cancers. AKT/protein kinase B (PKB) can inhibit the pro-apoptotic proteins and the Forkhead family of transcription factors, which upregulate other pro-apoptotic factors, for instance, Fas-ligand (FasL). Consequently, an overactivated PI3K/AKT/mTOR pathway is associated with increased resistance to apoptosis [28]. Additionally, dysregulation of this pathway can lead to uncontrolled cell growth, invasion, metastasis, and resistance to the therapy. Therefore, inhibitors of this pathway, such as mTOR inhibitors, have shown promise in clinical trials and this point of view can lead to exploring more agents that target the PI3K/AKT/mTOR pathway and act as effective anticancer therapeutics [22].

Regarding oxidative stress, the previous literature reported that under reasonable conditions of increased levels of ROS, AKT is activated. Indeed, the activated AKT can also increase the production of ROS. Hence, PI3K/AKT/mTOR can regulate oxidative stress with a complex effect on the redox homeostasis [23]. However, cancerous cells exhibit imbalanced redox homeostasis where the antioxidant mechanisms are overwhelmed and the over-proliferation of the cells is greatly associated with high ROS production [29]. Unfortunately, the produced ROS can modify the expression of the P53 tumor suppressor gene, resulting in evading apoptosis. Oxidative stress can also contribute to tumorigenesis by triggering DNA damage, mutations, protein dysfunction, and lipid peroxidation [30]. Consistent with these outcomes, our results indicate that the untreated group showed a remarkable upregulation of PI3K, p-AKT, and p-mTOR, along with downregulation of the antioxidant SOD and GPX4, as well as decreased levels of GSH and MDA. This imbalanced microenvironment was reflected in a remarkable increase in tumor volume.

In contrast, treatment of the mice with *C. media* extract reversed these findings with a gradual reduction in the tumor volume and a significant tumor inhibition rate, being higher in the case of the higher dose (200 mg/kg). Similarly, preceding studies have revealed the antioxidant effect of different *Cycas* species, which could be due to the flavonoid and polyphenolic compounds [10,11,13]. It was also reported that *Cycas* species exert their antioxidant effect through inhibition of microRNA216a, which is a key activator for the PI3K/AKT/mTOR pathway [31], resulting in a reduced level of MDA and increased level of GPX in a rat model of brain damage and pancreatitis [32]. Our results are in harmony with these conclusions and these favorable effects may be attributed to the commonly detected phytochemicals in our extract including dicarboxylic acids derivatives, terpenes, pyroglutamic acid, salicylate derivatives, essential and long-chain fatty acids, and mainly flavonoids.

Dicarboxylic acids represent antioxidant properties by acting as free radical scavengers, which are thought to be mediated through the donation of electrons or hydrogen atoms to the free radicals; thereby, neutralizing their reactivity and preventing them from causing oxidative damage to cells and tissues. Dicarboxylic acids can also regenerate some antioxidants and stimulate the activity of antioxidant enzymes such as SOD and GPX4 [33]. Another study has reported the inhibitory effect of dicarboxylic acids on the PI3K/AKT/mTOR pathway, thus, exerting antioxidant activity [34]. Terpenes also exhibited modulatory action on the PI3K/Akt/mTOR pathway by inhibiting its activity, leading to apoptosis and inhibition of cellular proliferation in malignant melanoma [35] and breast cancer cells [36]. In alignment with our findings, pyroglutamic acid previously exhibited antiproliferative activity and induced apoptosis on HeLa cervical cancer cells and K562 leukemia cells [37]. The antioxidant activity of pyroglutamic acid has been reported to be mediated through free radical scavenging and metal ion chelation [38].

Salicylate derivatives also showed inhibitory action for the PI3K/AKT/mTOR pathway, which may contribute to its anti-carcinogenic properties in the context of colorectal tumorigenesis [39]. The antioxidant mechanism of salicylates was suggested to involve several pathways and mechanisms including the scavenging of free radicals, inhibition of ROS production, induction of antioxidant enzymes, and inhibition of lipid peroxidation [40]. Essential and long-chain fatty acids were also detected in our extract as significant constituents. Preceding studies showed that α-linolenic acid and other omega-3 fatty acids demonstrated a repressive effect on the PI3K/AKT/mTOR pathway in hepatic steatosis [41] and prostatic cancer [42]. Long-chain fatty acids showed antioxidant properties mediated through upregulation of the antioxidant enzymes. This antioxidant activity is beneficial for normal cells and can disrupt the antioxidant balance in cancer cells, making them more susceptible to apoptosis [43]. Flavonoids were also chief compounds in our extract and there is no doubt that flavonoids exhibited antitumor and strong antioxidant activity over recent years. One of the proposed mechanisms through which flavonoids exert their anticancer effect was the inhibition of the PI3K/AKT/mTOR pathway in different cancerous cells [44,45,46]. The antioxidant effect was suggested to be facilitated through free radical scavenging, metal chelation, inhibition of enzymes involved in the generation of ROS, and induction of antioxidant enzymes [47,48].

Regarding the toxicity profile of our extract, the mice treated with *C. media* extract showed significantly lower levels of hepatic, renal, and cardiac biomarkers compared to the untreated group. Our results are in line with previously published data about the safety profile of different *Cycas* species including liver enzymes [32,49,50], kidney markers [32,51], and heart enzymes [52]. These findings suggest that *C. media* extract could be a potential antitumor agent with a good safety profile.

Different infections are frequently prevented and treated using many plant extracts that are frequently utilized traditionally. Such plant extracts often possess great advantages such as potency, safety, good bioavailability, and affordability [53]. Here, we studied the antimicrobial potential of *C. media* against *S. aureus* isolates from patients suffering from cancer. Remarkably, *C. media* displayed antibacterial potential against *S. aureus* isolates with MICs of 32 to 128 µg/mL. We explored its influence on the growth kinetics of *S. aureus* isolates. *C. media* induced a noteworthy bacterial growth retardation. Moreover, it caused a substantial weakening of the membrane integrity of *S. aureus* cells. The particular mechanism of action of *C. media* on *S. aureus* isolates requires further deep studies in the near future. However, our study has drawn attention to *C. media* as a probable antibacterial drug with an inhibitory effect on the physiologic bacterial growth. Also, the effect of *C. media* on the bacterial membrane has a role in its antibacterial action [54].

## 4. Materials and Methods

### 4.1. Collection, Drying, and Extraction of C. media

The leaves of *C. media* were collected in February 2021 from El Abd Garden in Giza City. The plant was dried and powdered to obtain 0.6 Kg dry weight. It was revealed by Dr. Esraa Ammar, Faculty of Science, Tanta University, and researcher Rabea Sharawy, Agronomist and palm researcher. A voucher specimen (PG-G-W-028) was kept at Tanta University in the herbarium of Tanta University. Methanol was utilised as an extracting solvent and was mixed with the powder. Extraction was performed thrice (4 L each), then the concentration of the solvent was performed by rotary evaporator under vacuum to obtain 29.6 g of total plant extract residue [15].

The LC–MS/MS analysis of *C. media* extract was achieved adopting methods reported before [55,56]. The negative electrospray ionization was employed to identify the phytoconstituents of the *C. media* extract [57,58].

### 4.2. Cell Line Culture

HepG2 cells were brought from VACSERA (Giza, Egypt). The provider of the culture reagents was Lonza (Walkersville, MD, USA). The cells were maintained at carbon dioxide incubator at 37 °C.

### 4.3. MTT Cytotoxicity Assay

Thermo Fisher Scientific^®^ (Waltham, MA, USA) provided the MTT kit, which was utilized to test in vitro cytotoxicity. A 96-well plate containing ten percent fetal bovine serum, one percent L-glutamine, one percent penicillin/streptomycin, and RPMI were used to culture 5 × 10^5^ HepG2 cells. After that, the cells were incubated for 24 h. The cells were treated with several concentrations of *C. media* extract (ranging from 2 to 0.0625 mg/mL dissolved in the culture media) for a whole day. To assess the viability of the cells, 20 μL of MTT reagent was added to each well and incubated for 4 h. Using a Tecan, Austria microplate reader, the absorbance of each well was measured at 570 nm following the addition of 200 µL of dimethyl sulfoxide (DMSO). Cell viability % = At/Ac × 100 was the formula used to calculate cell viability, where At is the absorbance of the extract and Ac is the absorbance of the control, Then, IC_50_ was determined as previously revealed [59].

### 4.4. Cell Cycle Analysis

It was evaluated using flow cytometry through incubation with propidium iodide (PI). After 24 h of incubation with 500 µg/mL *C. media* extract, the cells were harvested and washed with phosphate-buffered saline, followed by fixation with 70% ethanol. The cells were then collected and washed again followed by centrifugation at 1200 rpm for five minutes. Afterward, the cells were maintained in PBS with 50 μg/mL of RNase for two hours, incubated with 25 μg/mL of PI stain [60,61], and analyzed by flow cytometer (Becton Dickinson, US). The obtained data of cell cycle phases were analyzed using BD FACSDiva ™ software v9.0. The used reagents were from Merck KGAA (Darmstadt, Germany).

### 4.5. Experimental Animals and Study Design

Research Ethical Committee, Faculty of Pharmacy, Tanta University has approved the proposal of the study (TP/RE/7/24 p-01). For this investigation, forty female albino mice, 7–8 weeks and weighing 20–30 gm were utilized. The mice had unrestricted access to regular pellet food and water. The mice were given subcutaneous implants of 1 × 10^6^ EAC cells in their right thigh to induce the model. After 12 days, solid tumors that were palpable and around 100 mm^3^ in size were developed to form SEC [62,63]. Besides the normal control group, the diseased mice were split up into three groups to have a total of four groups in this study (*n* = 10). The vehicle was given to the normal and tumor control groups. For 16 days, the third and the fourth groups, designated as *C. media* 100 and *C. media* 200 were received daily oral dose of 100 mg/kg and 200 mg/kg of *C. media* methanolic extract, respectively. The extract was dissolved using a mixture of DMSO, saline, and propylene glycol (fifty percent DMSO, thirty percent saline, and twenty percent PEG). The size of the tumor was measured daily starting from the twelfth day using a vernier caliper (Tricle, Shanghai, China). The volume was calculated with the formula: 0.5 x (AB^2^), where A represents the minor axis length and B represents the major axis length [64,65]. The TIR was determined using the formula [52]:

$$\mathrm{TIR}=\frac{\mathrm{average}\;\mathrm{tumor}\;\mathrm{volume}\;\mathrm{of}\;\mathrm{the}\;\mathrm{control}\;\mathrm{group}-\mathrm{average}\;\mathrm{tumor}\;\mathrm{volume}\;\mathrm{of}\;\mathrm{the}\;\mathrm{treated}\;\mathrm{group}}{\mathrm{average}\;\mathrm{tumor}\;\mathrm{volume}\;\mathrm{of}\;\mathrm{the}\;\mathrm{control}\;\mathrm{group}}\times 100$$

Blood samples were taken on day twenty-eight following the administration of halothane as an anesthetic. The sera were isolated by a centrifuge (Sigma 3–30KS, Sigma, Osterode am Harz, Germany). After being extracted, the tumors were weighed, washed with saline, and then separated into several sections and they were kept for further examination in storage at −80 °C.

### 4.6. Determination of Biochemical Parameters

#### 4.6.1. Toxicity Profile

An ELISA Kit designed specifically for mice (abcam, Waltham, MA, USA) was used to measure alanine aminotransferase (ALT) and aspartate aminotransferase (AST) levels in the serum (Catalog nos. ab282882 and ab263882, respectively). A mouse-specific ELISA Kits (Catalog no. E-EL-M1801, E-EL-M0355, E-BC-K183-S, and E-EL-0058, respectively) were used to assess the quantities of cardiac troponin (cTnT), creatin kinase (CK-MB), urea, and creatinine in serum (Elabscience, Houston, TX, USA).

#### 4.6.2. Evaluation of Oxidative Stress

Following homogenization of the tumor tissue, the samples were centrifuged at 4000 rpm for 15 min at 4 °C. Then, glutathione (GSH) and malondialdehyde (MDA) levels were determined in the resulting supernatants (Catalog no. E-BC-K030-S and E-BC-K025-S, Elabscience, Houston, TX, USA), respectively, in accordance with the manufacturer’s instructions.

#### 4.6.3. Western Blot Assay for PI3K, p-AkT and p-mTOR

RIPA lysis buffer (Bio BASIC INC., Markham, ON, Canada) was used to recover all of the proteins from the tumor tissues. To determine the number of proteins in the supernatant, the lysate was centrifuged at 16,000× *g* for 30 min using a Bradford assay kit (BIO BASIC INC., Markham, ON, Canada). The extracted protein was separated using 12% SDS-PAGE gel electrophoresis, afterward transmitted to polyvinylidene difluoride membranes (Bio-Rad Laboratories, Hercules, CA, USA). Following the transfer of protein, the membranes underwent washing, and then the Pierce TM clear milk blocking solution (Thermo Fisher Scientific, Waltham, MA, USA) was employed to block the membranes for 30 min. Afterward, diluted primary antibodies were added, and the membranes were incubated for 16 h at 4 °C. After being incubated with secondary antibodies. Antibodies used for this study were as follows: PI3K (p110α, #4249), p-AKT (S473, #9271), and p-mTOR (S2448, #2971), were purchased from Cell Signaling Technology (Cell Signaling Technology, Danvers, MA, USA.), and anti-β-actin antibody was purchased from Sigma (Sigma-Aldrich, St. Louis, MO, USA). Blots were repeatedly rinsed in Tris-buffered saline with 0.05 percent Tween. Using the ECL-Western blot substrate, bands were seen. Following β-actin normalization, the target proteins’ band intensities were measured on the ChemiDoc MP imager in comparison to control samples.

#### 4.6.4. Gene Expression Analysis

Qiagen miR-Neasy Mini kit (Cat. No. 217004) (Qiagen, Hilden, Germany) was used for RNA extraction from the tissue. To make cDNAs from the extracted RNAs, Easy-Script^®^ First-Strand cDNA Synthesis SuperMiX (TransGen Biotech Co., Beijing, China) was utilized. Then, using QuantiTect^®^ SYBR^®^ Green PCR (QIAGEN, Hilden, Germany) and StepOnePlusTM Real-Time PCR equipment (Thermo Fisher Scientific, Waltham, MA, USA), the amplification step was carried out. Serving as the reference gene was β-actin. Relative copy number (RCN) was used to represent the expression levels of superoxide dismutase and glutathione peroxidase 4 (GPX4). The used primer sequences are illustrated in Table 3. The 20 μL PCR reaction consisted of 10 µL of SYBR Green PCR Master Mix, one microliter of each primer (10 μM) in addition to four microliters of cDNA and nuclease-free water. The 40-cycle PCR cycle starts with 15 s of 94 °C denaturation, 30 s of 50 °C annealing, and one minute of 65 °C extension.

### 4.7. Bacteria

Eighteen *S. aureus* isolates from cancer patients at Tanta University Hospital. The samples were attained for the routine laboratory diagnosis. Standard biochemical tests were employed for bacterial identification.

#### 4.7.1. Sensitivity to *C. media*

*C. media* antibacterial action was examined via cup plate assay as previously explained [66]. The performed wells in the Muller–Hilton agar plates, inoculated with bacterial suspension, were filled with 100 μL of the extract (1000 μg/mL), dimethyl sulfoxide (10%) as negative control, and vancomycin as positive control (reference drug).

#### 4.7.2. Minimum Inhibitory Concentration (MIC) of *C. media* Extract

The values of MICs of *C. media* were recorded by broth microdilution method as previously explained [67] as the lowest concentration that triggered a complete bacterial growth absence after incubation. The microtitration plates had a positive control (bacterial suspension) as well as a negative control (broth). Vancomycin was employed as a reference drug.

#### 4.7.3. Influence on the Growth of *S. aureus* Isolates

It was investigated at 0.5 MIC values by recording the optical density (OD) of *S. aureus* isolates with and without *C. media* at 620 nm by UV–Vis spectrophotometer (SHIMADZU, Kyoto, Japan). The time intervals at which the OD values were detected were 0, 1, 3, 5, 7, 24 h. Finally, a curve was obtained between log OD_620_ and time (h). Vancomycin was employed as a reference drug.

#### 4.7.4. Influence on the Membrane Integrity of *S. aureus* Isolates

It was revealed with and without treatment with *C. media* (at 0.5 MIC). This was performed by observing the liberation of DNA and RNA from the cells [68]. After centrifuging the bacterial suspensions, the obtained pellets were resuspended in sodium chloride solution (0.5%) and the absorbance was recorded at 260 nm. Vancomycin was employed as a reference drug.

### 4.8. Statistics

GraphPad Prism, version 10.0.1 was utilized, employing ANOVA and Tukey’s post hoc test for significant differences. Results were considered significant at *p* < 0.05 and were revealed as mean ± standard deviation (SD).

## 5. Limitations of the Study and Future Prospectives

Our study demonstrated promising consequences regarding the anticancer, antioxidant, and antibacterial effects of *C. media* extract. Nonetheless, one limitation is the use of only one cancer and non-tumorigenic cell lines for cytotoxicity assessment; future studies should include additional cell lines to validate our findings. We also recommend isolating the most biologically active compounds and conducting further pharmacological screening with a range of higher extract concentrations. Additionally, performing RNA sequencing and determining the total protein levels to estimate the ratio of phosphorylated proteins would provide valuable insights for future research in this field.

## 6. Conclusions

*C. media* extract showed an antiproliferative effect against HepG2 cells. Moreover, it also demonstrated suppressive action on the PI3K/AKT/mTOR pathway and an antioxidant effect mediated through the upregulation of the antioxidant enzymes, SOD and GPX4 against an experimentally induced mammary carcinoma animal model. These beneficial outcomes suggest *C. media* to have a potential antitumor activity but further investigations are recommended. Also, *C. media* revealed antibacterial action against *S. aureus* isolates from cancer patients. It might be a solution for the antibiotic resistance in the pathogenic bacteria, particularly *S. aureus*. Therefore, future preclinical and clinical trials should be performed to evaluate its efficacy.

## Figures and Tables

**Table 1:** Metabolite profiling of *C. media* extract by LC–ESI–MS/MS analysis (negative mode ESI).

No.	Rt (min)	Precursor m/z	Error ppm	Name	Structure	Formula	Adduct Ion	MS/MS Spectrum	Ontology
1	1.10	117.0189	−1.1	Succinic Acid	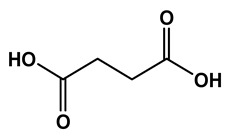	C_4_H_6_O_4_	[M-H]^-^	117.0180,99.0140, 75.3908,73.0291, 55.0200	Dicarboxylic acids and derivatives
2	1.10	265.0700	10	1,4-dihydroxy-6,6,9a-trimethyl-4,5,5a,7,8,9-hexahydro-1H-benzo[e][2]benzofuran-3-one	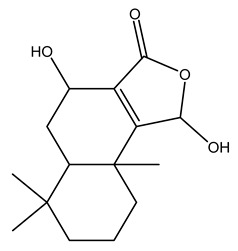	C_15_H_22_O_4_	[M-H]^-^	265.1468, 264.2965, 112.9858, 96.9597, 79.9573	Naphthofurans
3	1.16	128.0345	−1.6	Pyroglutamic Acid	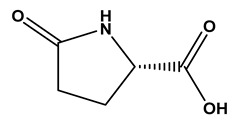	C_5_H_7_NO_3_	[M-H]^-^	128.0339, 85.9955, 82.0320	Alpha amino acids and derivatives
4	1.27	123.0451	−0.5	Salicylic Alcohol	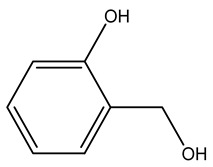	C_7_H_8_O_2_	[M-H]^-^	123.0446, 122.0363, 95.0104, 77.0429, 67.0158	Benzyl alcohols
5	1.32	181.0710	−0.7	Mannitol	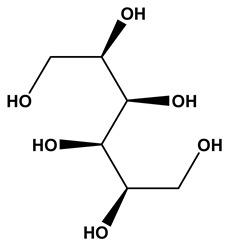	C_6_H_14_O_6_	[M-H]^-^	181.0716, 163.0609, 119.0347, 113.0240, 101.0240, 89.0251, 73.0313, 71.0134, 59.0140	Sugar alcohols
6	1.44	269.0869	1	Dalbergione, 4-Methoxy-4′-Hydroxy-	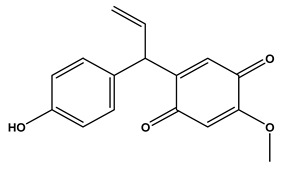	C_16_H_14_O_4_	[M-H]^-^	269.0829, 255.0632, 254.0579, 253.0507, 237.0544, 225.0539, 92.9273	Dalbergiones
7	5.93	121.0293	0.4	3-Hydroxybenzaldehyde	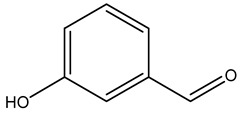	C_7_H_6_O_2_	[M-H]^-^	121.0298, 120.0249, 108.0225, 93.0362, 92.0260, 91.0163	Hydroxybenzaldehydes
8	6.85	541.2435	−0.7	methyl (2S,3R,4S)-3-ethenyl-4-[2-(3,4,5-trihydroxybenzoyl)oxyethyl]-2-[3,4,5-trihydroxy-6-(hydroxymethyl)oxan-2-yl]oxy-3,4-dihydro-2H-pyran-5-carboxylate	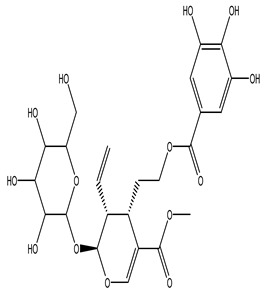	C_24_H_30_O_14_	[M-H]^-^	541.1399, 473.1713, 405.1728, 347.2330, 337.1846, 279.2397, 170.9432, 102.9612	Terpene glycosides
9	6.93	227.0830	0.3	Aspernigrin A	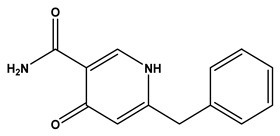	C_13_H_12_N_2_O_2_	[M-H]^-^	227.0827, 210.0779, 209.0721, 184.0767, 143.0517, 115.0550, 109.0179, 65.9984	Nicotinamides
10	7.24	447.0951	0.9	Kaempferol-3-O-glucoside	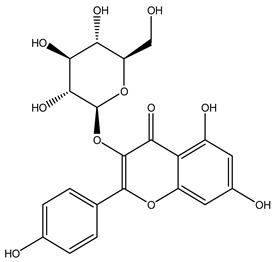	C_21_H_20_O_11_	[M-H]^-^	447.0933, 285.0426, 284.0321, 255.0317, 227.0353	Flavonoid-3-*O*-glycosides
11	7.33	195.9125	−0.3	3,5,6-Trichloro-2-pyridinol	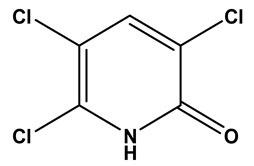	C_5_H_2_Cl_3_NO	[M-H]^-^	195.9134, 152.9770, 112.9862, 84.9911	Polyhalopyridines
12	7.71	431.0983	0.3	Apigetrin	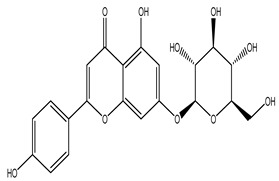	C_21_H_20_O_10_	[M-H]^-^	431.0984, 429.7303, 311.0560, 269.0454, 268.0374, 240.0424, 211.0428, 151.0095, 102.9557	Glycosyloxyflavone
13	7.81	287.0559	−0.5	Dihydrokaempferol	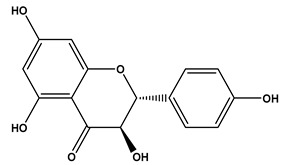	C_15_H_12_O_6_	[M-H]^-^	287.0569, 259.0610, 255.1236, 243.0660, 215.0712, 201.0556, 177.0559, 151.0042, 125.0243, 83.0138, 57.0346	Flavanone *O*-glycosides
14	7.88	327.1811	0.6	Spiculisporic Acid	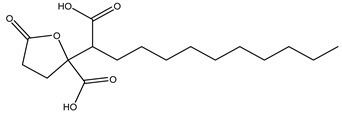	C_17_H_28_O_6_	[M-H]^-^	327.1796, 283.1905, 265.1798, 255.1962, 239.2008, 221.1908, 211.2069, 124.0161	Tricarboxylic acids and derivatives
15	7.96	433.1140	1.2	Engeletin	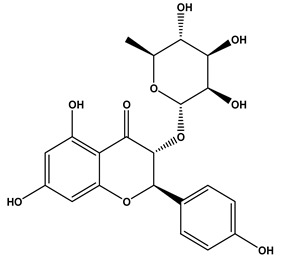	C_21_H_22_O_10_	[M-H]^-^	433.1170, 389.1336, 321.1472, 305.1732, 257.1591, 221.1964, 112.9841	flavonoids
16	9.70	293.1760	0.6	13-Oxo-9,11-octadecadienoic Acid	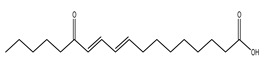	C_18_H_30_O_3_	[M-H]^-^	293.2109, 275.2029, 236.1047, 208.9180, 195.1355, 183.1387, 171.1040, 102.9558, 94.9244, 92.9231	Linoleic acid and derivatives
17	10.04	537.0833	0.9	Agathisflavone	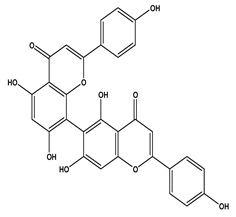	C_30_H_18_O_10_	[M-H]^-^	537.0814, 443.0404, 417.0619, 399.0517, 375.0504, 331.0621, 159.0447, 117.0342	Biflavonoids and polyflavonoids
18	10.09	537.0827	0.9	Amentoflavone	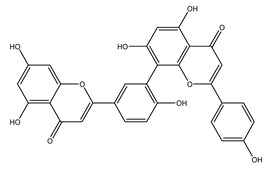	C_30_H_18_O_10_	[M-H]^-^	537.0814, 443.0404, 417.0619, 399.0517, 375.0504, 333.0403, 331.0621, 307.0619, 203.0348, 159.0447, 117.0342	Hydroxyflavone
19	10.27	357.1330	0.4	Matairesinol	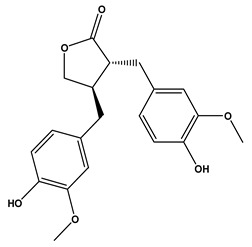	C_20_H_22_O_6_	[M-H]^-^	357.1361, 342.1190, 220.8766, 151.0404, 136.0181, 112.9855, 104.9535, 94.9245	Dibenzylbutyrolactone lignans
20	10.64	269.0452	0.2	Aloe-emodin	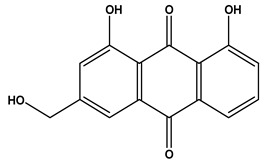	C_15_H_10_O_5_	[M-H]^-^	269.0456, 225.0597, 151.0040, 149.0234, 117.0343	Anthraquinones
21	11.67	551.0984	0.9	Bilobetin	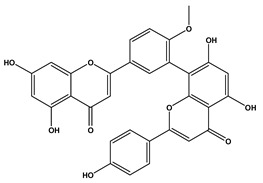	C_31_H_20_O_10_	[M-H]^-^	551.0982, 457.0592, 431.0785, 413.0678, 389.0669, 345.0790	Flavonoids
22	13.47	297.1535	10	Aurapten	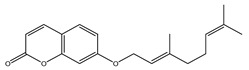	C_19_H_22_O_3_	[M-H]^-^	297.1526, 198.0321, 183.0132, 170.0015, 119.0497, 102.9575	Terpene lactones
23	15.17	565.1140	0.9	Isoginkgetin	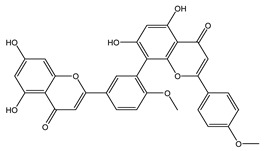	C_32_H_22_O_10_	[M-H]^-^	565.1138, 533.0879, 471.0707, 445.0926, 415.0446, 403.0810, 389.0672, 374.0446, 151.0037	Biflavonoid
24	16.27	419.2813	0.9	(1S,4S,5R,9S,10R,13R,14R)-14-hydroxy-5,9-dimethyl-14-{[(3-methylbutanoyl)oxy]methyl}tetracyclo [11.2.1.01,10.04,9]hexadecane-5-carboxylic acid	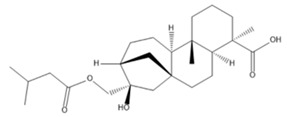	C_25_H_40_O_5_	[M-H]^-^	419.2803, 163.0418, 145.0301, 119.0511, 117.0335, 112.9823	Kaurane diterpenoids
25	16.45	297.2441	−0.4	Nephrosteranic acid	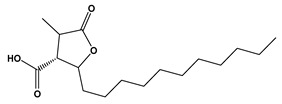	C_17_H_30_O_4_	[M-H]^-^	297.0773, 265.1813, 253.2146, 235.0776, 228.9046, 221.1908, 128.9603, 112.9846, 102.9567, 92.9283	lactone
26	18.13	581.1453	−1	Oliveriflavone	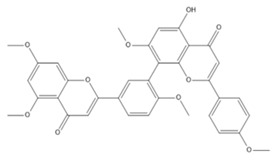	C_33_H_26_O_10_	[M-H]^-^	581.1444, 549.1185, 415.1158, 405.0975, 390.0802, 239.0690, 165.0206	Flavonoids
27	19.49	277.2180	0.6	Linolenic Acid	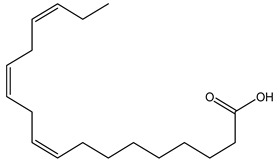	C_18_H_30_O_2_	[M-H]^-^	278.2198, 277.2172, 275.1969, 233.2271, 158.9255, 92.9304, 59.0142	Lineolic acid and derivatives
28	21.79	279.2711	1	Linoleic Acid	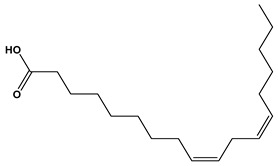	C_18_H_32_O_2_	[M-H]^-^	280.2361, 279.2330, 261.2231, 243.2082, 83.0574, 71.0126, 59.0137	Lineolic acid and derivatives
29	22.64	455.3549	0.8	Ursolic Acid	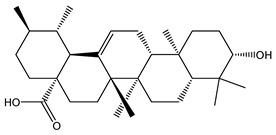	C_30_H_48_O_3_	[M-H]^-^	455.3532, 453.8744, 425.2720, 409.2371, 392.2421, 311.1802, 255.2156	Triterpenoids
30	23.44	255.2335	0.3	Palmitic Acid	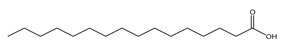	C_16_H_32_O_2_	[M-H]^-^	255.1387, 237.2210, 102.9529	Long-chain fatty acids
31	24.21	281.2494	1.9	Vaccenic Acid	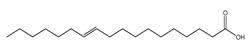	C_18_H_34_O_2_	[M-H]^-^	282.2533, 281.2491, 263.2374, 183.1453, 112.9852, 97.0656, 59.0188	Long-chain fatty acids
32	24.38	317.2484	0.5	Urushiol II	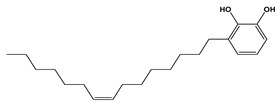	C_21_H_34_O_2_	[M-H]^-^	318.2525, 317.2500, 299.2453, 248.9615, 180.9711, 112.9863	Catechols
33	25.48	269.2500	0.5	Heptadecanoic Acid	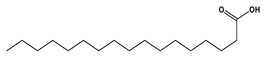	C_17_H_34_O_2_	[M-H]^-^	270.2537, 269.2488, 251.2356, 210.8343, 92.9280	Long-chain fatty acids

**Table 2:** The MICs of *C. media* against *S. aureus* isolates.

Bacterial Isolates	MICs (µg/mL)	Bacterial Isolates	MICs (µg/mL)
S1	64	S10	64
S2	64	S11	32
S3	128	S12	32
S4	32	S13	64
S5	64	S14	64
S6	128	S15	128
S7	64	S16	128
S8	32	S17	32
S9	64	S18	32

**Table 3:** Primer sequence.

	Forward	Reverse
SOD	TTGGCCGTACAATGGTGGTC	AGTTTAATGGTTTGAGGGTAGCA
GPX4	TACACCGAGATGAACGATCTG	ATTCTTGCCATTCTCCTGGT
β-actin	ACTATTGGCAACGAGCGGTT	AATGCCTGGGTACATGGTGG

SOD: superoxide dismutase, GPX4: glutathione peroxidase 4.

## Data Availability

Data are available upon reasonable request.
